# Supplementary figures and images for: Sepsis impedes EAE disease development and diminishes autoantigen-specific naive CD4 T cells
Source: eLife. 2020 Nov 16;9:e55800. doi: 10.7554/eLife.55800 (PMC7721438; doi:10.7554/eLife.55800)

## Slide 1
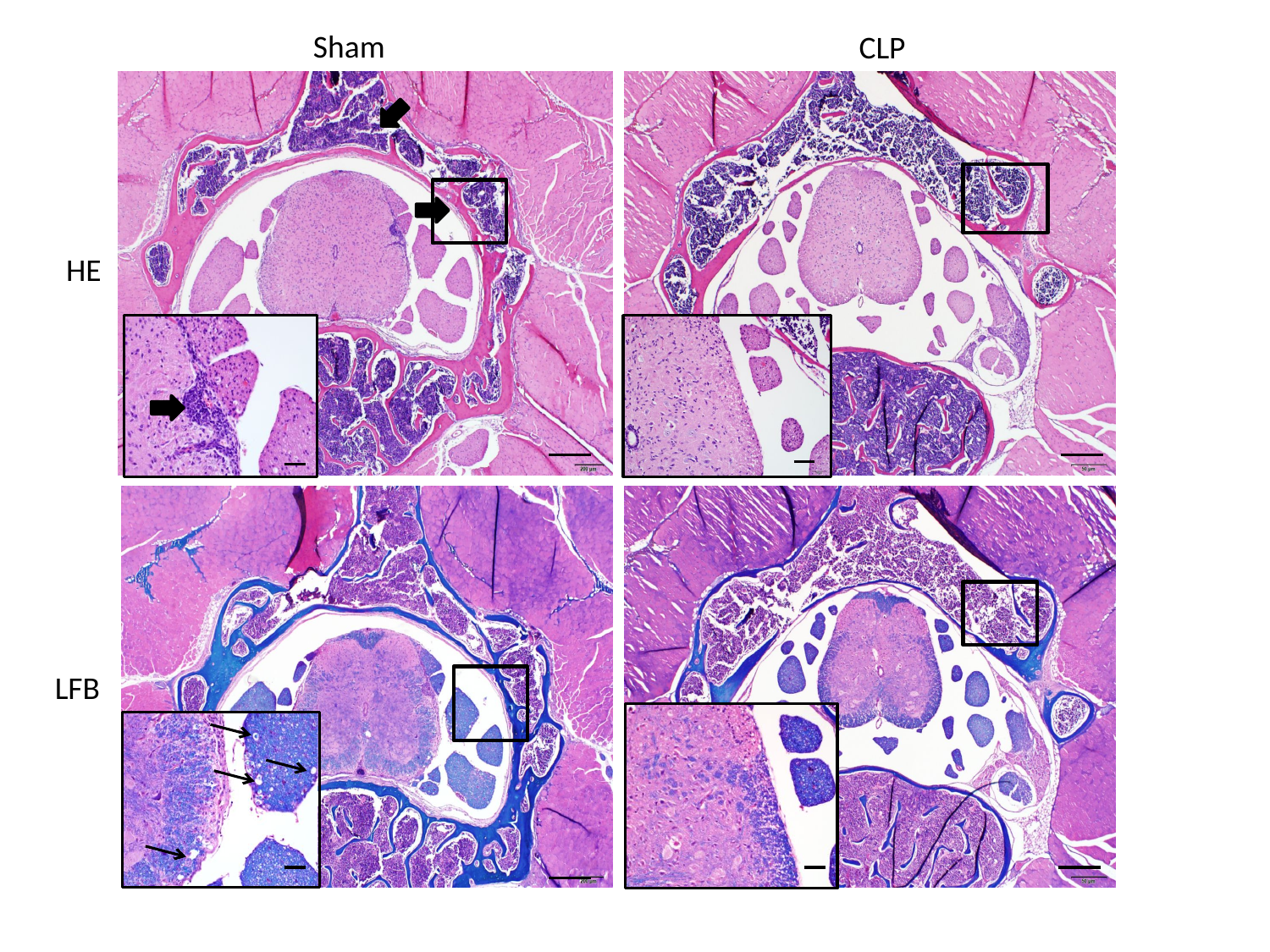

Sham
CLP
HE
LFB

Supplement: Figure 1—source data 2. [file elife-55800-fig1-data2.pptx]
